# To inhibit TrxR1 is to inactivate STAT3–Inhibition of TrxR1 enzymatic function by STAT3 small molecule inhibitors

**DOI:** 10.1016/j.redox.2020.101646

**Published:** 2020-07-17

**Authors:** Sander Busker, Brent Page, Elias S.J. Arnér

**Affiliations:** a Division of Biochemistry, Department of Medical Biochemistry and Biophysics, Karolinska Institutet, Stockholm, Sweden; b Department of Oncology-Pathology, Science for Life Laboratories, Karolinska Institutet, Stockholm, Sweden; c Faculty of Pharmaceutical Sciences, University of British Columbia, Vancouver, BC, Canada; d Department of Selenoprotein Research, National Institute of Oncology, Budapest, Hungary

**Keywords:** Signal transducer and activator of transcription 3, Thioredoxin reductase 1, Small molecule inhibitors, Electrophiles, Selenocysteine, Redox, STAT3, signal transducer and activator of transcription (STAT3), SH2-domain, Src Homology 2-domain, FPA, fluorescence polarization assay, EMSA, Electrophoretic mobility shift assay, ELISA, enzyme-linked immunosorbent assay, TrxR1, Thioredoxin Reductase 1, Sec, Selenocysteine, GSH, Glutathione, DTNB, 5,5′-dithiobis-(2-nitrobenzoic acid), T_m_, Melting temperature, BCA, Bicinchoninic acid, DSF, Differential scanning fluorimetry, SecTRAPs, Selenium compromised thioredoxin reductase-derived apoptotic proteins

## Abstract

The transcription factor STAT3 plays a key role in cancer and immunity, being widely explored as a potential drug target for the development of novel immunomodulatory or anticancer therapeutics. The mechanisms of small molecule-derived inhibition of STAT3 appear, however, to be more complex than initially perceived. Our recent discovery, that some novel STAT3 inhibitors were *bona fide* inhibitors of the cytosolic selenoprotein oxidoreductase TrxR1 (TXNRD1), led us to explore the effects of a wide array of previously described STAT3 inhibitors on TrxR1 function. We found that 17 out of 23 tested STAT3 small molecule inhibitors indeed inhibited purified TrxR1 at the reported concentrations yielding STAT3 inhibition. All tested compounds were electrophilic as shown by direct reactivities with GSH, and several were found to also be redox cycling substrates of TrxR1. Ten compounds previously shown to inhibit STAT3 were here found to irreversibly inhibit cellular TrxR1 activity (Auranofin, Stattic, 5,15-DPP, Galiellalactone, LLL12, Napabucasin, BP1-102, STA-21, S3I-201 and Degrasyn (WP1130)). Our findings suggest that targeting of TrxR1 may be a common feature for many small molecules that inhibit cellular STAT3 function. It is possible that prevention of STAT3 activation in cells by several small molecules classified as STAT3 inhibitors can be a downstream event following TrxR1 inhibition. Therefore, the relationship between TrxR1 and STAT3 should be considered when studying inhibition of either of these promising drug targets.

## Graphical abstract

**Figure:**
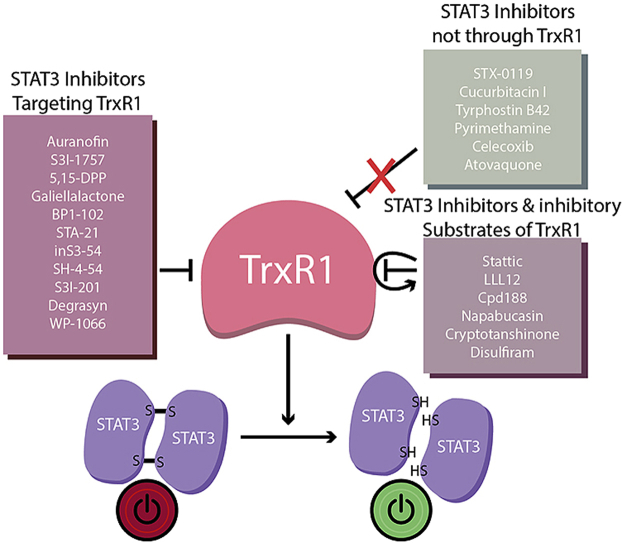

## 1 Introduction

Signal transducer and activator of transcription 3 (STAT3) is an important transcription factor for control of many processes in immunity and cancer [[1], [2], [3]]. In cancer, STAT3 supports oncogenesis, cell death resistance, anti-tumor immunity evasion, therapy resistance and additional critical processes for cancer development [2,[4], [5], [6], [7], [8]]. The STAT3 inhibitor field is hence a large and active research area for development of novel anticancer therapies, with many small molecules reported to inhibit STAT3 function in cells. Although their chemical structures vary, many of these compounds aim to bind the same site of STAT3, its Src Homology 2-domain (SH2-domain) [9,10]. Binding to the SH2 domain is believed to block STAT3 phosphorylation, dimerization and thereby its transcriptional function [11,12]. These small molecules were often confirmed to target STAT3 using biochemical assays, such as fluorescence polarization assay (FPA), electrophoretic mobility shift assay (EMSA) and enzyme-linked immunosorbent assay (ELISA) [10,12]. Additional cellular and *in vivo* experiments have also typically been employed to demonstrate inhibition of STAT3 phosphorylation, down-regulation of STAT3-dependent gene expression, or blockade of other STAT3-related cellular processes [9,13,14].

It has become clear that the biochemical and cellular effects of these inhibitors might also be caused by other mechanisms than direct binding to STAT3 in cells [15]. Recently, we found that several novel STAT3 inhibitors exerted their effects *via* covalent inhibition of Thioredoxin Reductase 1 (TrxR1, TXNRD1), rather than STAT3. In a cellular setting, inhibition of TrxR1 readily leads to oxidation of STAT3 cysteine residues, thereby impairing the transcriptional function of STAT3 [15,16]. Oxidation of STAT3 cysteine residues leads to the formation of inactive STAT3 multimeric or dimeric covalently bound disulfide-linked complexes [15,16]. TrxR1 is essential in order to prevent and reverse this oxidation, especially under conditions of oxidative stress. It is the driving enzyme for the cellular reductive systems that continuously keep these cysteine residues reduced in order to maintain STAT3 in a functional state [15,16]. Similar observations have been made with the Nrf2-Keap1 transcription factor system, oxidation of Keap1 cysteine residues are highly regulated by the reductive capacities of TrxR1 [17].

Several STAT3 inhibitors, including Stattic [14], S3I-201 [18], BP1-102 and SH-4-54 [19], contain electrophilic moieties that can form covalent adducts with cysteine residues on purified STAT3 protein, as well as other proteins [[20], [21], [22]]. These inhibitors may be especially reactive with TrxR1 due to its crucial and highly nucleophilic selenocysteine (Sec) residue in the active site of the enzyme [15,23]. Stattic was furthermore recently found to target the TrxR1 orthologue of *Schistosoma mansoni*, again likely by binding directly to its Sec residue [24]. In addition, a potent inhibitor of TrxR1, Auranofin, which is clinically approved for treatment of rheumatoid arthritis and currently in trials for anticancer treatment, was also shown to potently inhibit Interleukin-6 dependent JAK1/STAT3 phosphorylation [25].

These experiments have clearly suggested direct links between TrxR1 targeting and a number of different STAT3 inhibitors. TrxR1 is the cytosolic isoenzyme of the TrxR family. Using NADPH it propels the many functions of the reductive thioredoxin system, important for control of redox regulation, antioxidant defense and for general disulfide reduction in cells [[26], [27], [28]]. TrxR1 is very sensitive to inhibition by electrophiles due to its highly reactive, surface-exposed Sec residue that is essential for its reductive capacities [29], typically being 1000-fold more reactive than a cysteine residue [12,13,30]. Therefore, an array of chemotherapeutics are known as inhibitors of TrxR1 and, moreover, due to its importance for cancer cell survival, TrxR1 is a promising target on its own for novel anticancer therapeutics [[31], [32], [33], [34], [35], [36], [37]].

**Fig. 1:**
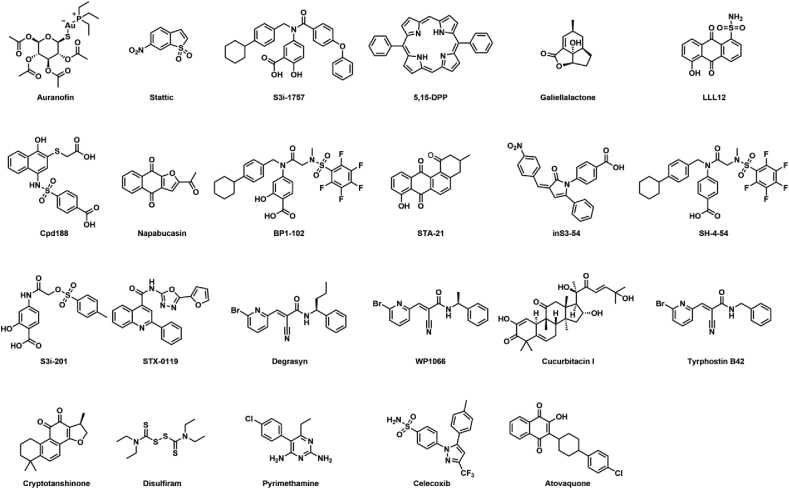
**Chemical structures of explored small molecule STAT3 inhibitors.** Compounds S3I-1757, BP1-102, SH-4-54 and S3I-201 all contain the same scaffold. LLL12, Napabucasin, STA-21, Cryptotanshinone and Atovaquone all contain a quinone structure. See Table 1 for further details.

To further investigate the possible targeting of TrxR1 by additional STAT3 inhibitors, we here explored a broad range of 23 different small molecules that have all been described in the literature as STAT3 inhibitors (Table 1, Fig. 1). Auranofin, Stattic, Galiellalactone, BP1-102, SH-4-54, S3I-201 and Degrasyn (WP1130) were previously shown to possess electrophilic properties [[20], [21], [22],[38], [39], [40]]. Degrasyn, WP1066 and Tyrphostin B42 are the only reported JAK inhibitors included in this study, containing the same backbone with the same electrophilic group (Fig. 1). Disulfiram, Pyrimethamine and Atovaquone are clinical drugs to treat chronic alcoholism, toxoplasmosis and pneumocystis pneumonia, respectively [[41], [42], [43]]. They were also reported to inhibit STAT3 (Table 1), but their exact mechanisms of STAT3 inhibition remain unknown. Assessing these 23 compounds for TrxR1 targeting, we found that 17 inhibited TrxR1 *in vitro* at concentrations in range of their reported STAT3-inhibitory concentrations with 6 compounds also being redox cycling substrates of TrxR1, all compounds interacted directly with reduced glutathione (GSH), and 10 of the compounds significantly inhibited cellular TrxR1 activity. The results suggest that targeting of TrxR1 may be a shared feature for several STAT3 inhibitors.

**Table 1:** Explored previously reported STAT3 inhibitors, their reported cellular targets and which methods were utilized to assess STAT3 inhibition.

Compound^a^	Reported targets	STAT3 Inhibitory concentration	Biochemical methods	Cellular methods	In vivo	Ref.
Auranofin	TrxR1/STAT3 (Covalent)	2 μM	EMSA	pSTAT3, Nuc, Gene	–	[25]
Stattic	STAT3	10–20 μM	FPA, EMSA	pSTAT3, Nuc	–	[14]
S3I-1757	STAT3	100 μM	FPA, ELISA	Nuc, Luci, Gene, Dim	–	[57]
5,15-DPP	STAT3 SH2-domain	20–50 μM	AlphaScreen, ELISA	pSTAT3, Nuc, ChIP	–	[58]
Galiellalactone	STAT3 (Covalent)	10 μM	EMSA	Luci, CPD	–	[39]
LLL12^c^	STAT3	5–10 μM	–	pSTAT3, Nuc, Gene	Xeno	[59]
Cpd188	STAT3 SH2-domain	100 μM	SPR	pSTAT3, Nuc	–	[54]
Napabucasin^c^	STAT3, NQO1 substrate	0.5–2 μM	–	pSTAT3	–	[60]
BP1-102	STAT3 SH2-domain	5–20 μM	EMSA	pSTAT3, Gene.	Xeno	[19]
STA-21^c^	STAT3 SH2-domain	20–30 μM	EMSA	Nuc, Luci, Gene, Dim	–	[61]
inS3-54	STAT3 DNA binding-domain	20–25 μM	EMSA	pSTAT3, Luci, CPD, Gene, ChIP	–	[62]
SH-4-54	STAT3/STAT5 SH2-domain	0.5–5 μM	SPR	pSTAT3, Gene	Xeno	[63]
S3I-201	STAT3 SH2-domain	30–100 μM	EMSA	pSTAT3, Luci, Gene, Dim	Xeno	[18]
STX-0119	STAT3	20–100 μM	–	pSTAT3^b^, Luci, Gene, Dim, ChIP	Xeno	[64]
Degrasyn (WP1130)	JAK2 (Covalent), STAT3	5–50 μM	–	pSTAT3	–	[40]
WP1066	JAK2/STAT3	10 μM	–	pSTAT3, Nuc, Gene	Xeno	[65]
Cucurbitacin I (JSI-124)	STAT3	10 μM	EMSA	pSTAT3, Luci	Xeno	[66]
Tyrphostin B42 (AG490)	JAK2, STAT3	100 μM	EMSA	pSTAT3	–	[67]
Cryptotanshinone^c^	STAT3 SH2-domain, 11β-HSD1, NQO1 substrate	7–10 μM	EMSA	pSTAT3, Luci, Gene, Dim	–	[[68], [69], [70]]
Disulfiram	ALDH1A1, STAT3	0.5 μM (+CuCl_2_)	–	pSTAT3, Nuc, Gene	–	[41]
Pyrimethamine	DHFR, STAT3	10–100 μM	–	pSTAT3, Luci	–	[42,71]
Celecoxib	STAT3 SH2-domain, COX-2	50 μM	–	pSTAT3	–	[72]
Atovaquone^c^	parasitic ETC, STAT3	15–20 μM	–	pSTAT3, Luci, Gene.	Xeno	[43,55]

Abbreviations: fluorescence polarization assay (FPA), electrophoretic mobility shift assay (EMSA), Surface Plasmon Resonance (SPR), STAT3 phosphorylation (pSTAT3), STAT3-driven luciferase transcription (Luci), Activation-driven nuclear translocation (Nuc), STAT3-driven target gene expression (Gene), STAT3 pull-down with compound (CPD), STAT3 dimerization (Dim), Promoter binding (ChIP), Mouse xenografts (Xeno).

a See Fig. 1 for compound structures.

b No discernable inhibitory effect of compound on readout.

c Contains a quinone in its chemical structure.

## 2 Material and methods

### 2.1 Enzymes, reagents and small molecule inhibitors

TrxR1 enzyme was recombinantly produced with high Sec contents in a specific Release Factor 1-depleted *E. coli* strain and purified as previously described [44]. NADPH and GSH were purchased from PanReac AppliChem, 5,5′-dithiobis-(2-nitrobenzoic acid) (DTNB), Tris base, HCl, EDTA and Sypro Orange™ were purchased from Sigma-Aldrich. NADPH was always freshly dissolved in TE buffer. DTNB was dissolved in ethanol.

Auranofin was purchased from Enzo Life Sciences. Stattic was purchased from Selleck Chemicals. 5,15-DPP and inS3-54 were purchased from Sigma-Aldrich. Galiellalactone was purchased from Tocris. SH-4-54 was purchased from Cayman Chemicals. LLL12, Napabuscasin, BP1-102, S3I-201, STX-0119, Degrasyn, WP-1066, Cucurbitacin I, Tyrphostin B42, Cryptotanshinone and Pyrimethamine were kindly provided by Dorian Cheff from the Chemical Genomics Center of the National Center for Advancing Translational Sciences (NCGC, NCATS, NIH, MD, USA). S3I-1757, Cpd188, STA-21, Disulfiram, Celecoxib and Atovaquone were kindly provided by Sanaz Attarha. All compounds were dissolved in DMSO excluding Auranofin, which was dissolved in ethanol. All compounds were stored at −20 °C.

### 2.2 Cell culture conditions

A549 cells were grown at 37 °C and 5% CO_2_ in RPMI-1640 medium containing 10% FBS, 2 mM l-glutamine and penicillin/streptomycin (100 μg/mL). Media was additionally supplemented with 100 nM sodium selenite for at least 72 h before commencement of any experiments to allow for stable and saturated selenoprotein expression. Medium and other cell culture reagents were purchased from Gibco (Thermo Scientific). DMSO concentrations did not exceed 1% final concentration, except for 5,15-DPP which was at 2,5% final concentration due to poor solubility of the compound in DMSO.

### 2.3 Recombinant TrxR1 enzyme activity assay

Sec-dependent TrxR1 activity was assessed as previously described [34]. In short, using a direct TrxR1-linked DTNB reduction assay, compounds were incubated with TrxR1 in a reaction containing 12,5 nM TrxR1, 0.1 mg/mL BSA, with 250 μM NADPH in a volume of 80 μL in 384 well plates in TE buffer (50 mM Tris-HCl, 2 mM EDTA, pH 7.5). After 40 min 20 μL of DTNB was added to a final concentration of 1 mM DTNB, and TrxR1-dependent TNB production was measured as the time dependent increase in absorbance at 412 nm during 6 min using a Tecan Infinite M200 Pro. DMSO concentrations were kept constant for each compound concentration at a final concentration of 2%, except 100 μM 5,15-DPP, where DMSO was 5% due to poor solubility of the compound.

### 2.4 Glutathione reactivity assay

Similar to previously reported methods [15], 175 μM compound was incubated with 175 μM GSH in TE buffer. At 0, 1, 2, 4, 6 and 24 h 10 μL of the reaction was mixed with 100 μL 1 mM DTNB, in order to assess the concentration of reduced GSH remaining in the reaction. Released TNB was measured as the resulting absorbance at 412 nm using a Tecan Infinite M200 Pro. For each measured timepoint, DMSO containing reactions were set to 100%, excluding Auranofin which was compared to ethanol containing reactions, after background subtraction. At 175 μM 5,15-DPP, LLL12, Cpd188 and Atovaquone interfered with 412 nm absorbance and respective background subtraction was performed using reactions containing solely 175 μM of each compound in TE buffer.

### 2.5 TrxR1 differential scanning fluorimetry assay

Similar to previously reported methods [12], TrxR1 was used at 1 μM together with NADPH at 500 μM and Sypro Orange™ at “5x” final concentration. All DSF assays were performed in TE buffer in a total volume of 20 μL per reaction. Compounds at indicated concentrations were added in DMSO at a final concentration of 2%. Reactions were run in 96-well plates on a PikoReal Real-Time PCR System (Thermo Scientific), with fluorescence measured using Channel 5 and 475–500 nm excitation range, and a 520–590 nm emission range. Reactions were heated gradually from 30 to 85 °C with increments of 1 °C per minute. Raw fluorescence signal was normalized to maximum values, followed by curve fitting in GraphPad Prims to a Boltzman sigmoidal curve. Addition of some compounds led to data points that did not properly fit the Boltzman sigmoidal curve, therefore some temperature values were excluded in order to retrieve a representable melting temperature (T_m_).

### 2.6 Kinetic characterization of STAT3 inhibitors as redox cycling TrxR1 substrates

Enzymatic activity of TrxR1 was determined through NADPH consumption upon addition of STAT3 inhibitors within the course of 90 min. Compounds were incubated in an 80 μL reaction containing 1 μM TrxR1, 300 μM NADPH and 0,1 mg/mL BSA in TE buffer with a final DMSO concentration of 1%. Time-dependent changes in NADPH concentrations were continuously measured at 340 nm absorbance using a Tecan Infinite F200 Pro fitted with 340 nm filter (10 nm range). The amount of NADPH was calculated using a NADPH standard curve from 0 to 400 μM. Experiments were performed in 384 well plates in triplicate.

### 2.7 Recombinant TrxR1 enzyme covalent inhibitor characterization

Sec-dependent TrxR1 activity was assessed as described above. Compounds were however incubated in a reaction containing 315 nM TrxR1, 0.1 mg/mL BSA, with 200 μM NADPH in a volume of 200 μL in 96 well plates in TE buffer a final DMSO concentration of 5%. After 40 min, 10 μL reaction mixture was mixed with 190 μL of DTNB and NADPH at a final concentration of 1 mM DTNB and 200 μM NADPH. TrxR1 activity was assessed by TNB production measured as the time dependent increase in absorbance at 412 nm for 6 min. Subsequently, 160 μL reaction mixture was desalted using Zeba™ Spin Desalting Columns 40 K (Thermo Scientific), whereupon 50 μL desalted enzyme was mixed with 150 μL of DTNB and NADPH to a final concentration of 1 mM DTNB and 200 μM NADPH. Then TNB production was again measured as the time dependent increase in absorbance at 412 nm for 1,5 min using a Tecan Infinite F200 Pro fitted with 405 nm filter (10 nm range).

### 2.8 Cellular thioredoxin reductase activity assay

Following a protocol reported previously [34], A549 cells were seeded at a density of 500 000 cells per well in 6-well plates. After 24 h, compounds were added at indicated concentrations with a final concentration between 0.01% and 0.5% of DMSO, and incubated at 37 °C for 4 h. Cells were then lysed for 10 min in TE buffer (50 mM Tris-HCl, 2 mM EDTA, pH 7.5), containing 1% NP-40, Phosphatase and Protease inhibitor cocktails (Roche). Protein concentrations in cleared supernatants, after 10 min centrifugation at 10,000 g, were determined using the bicinchoninic acid (BCA) assay. TrxR1 activity was determined using an insulin endpoint assay coupled with Trx1, with 5 μg protein lysate incubated with 0.16 mM insulin, 0.33 mM NADPH and 16 μM Trx1 in TE buffer. Samples were incubated at room temperature for 30 min followed by addition of 7.2 M Guanidine-HCl (pH 8.0) with 2.5 mM DTNB, and absorbance at 412 nm was measured using a Tecan Infinite M200 Pro. The activity of DMSO-treated control cells was put to 100%, after background subtraction.

### 2.9 Statistical analyses

Statistical analyses were performed in GraphPad Prism version 8.3.0. One-way ANOVA's were used to generate p-values, with a Bonferroni Multiple comparisons test. P-values are displayed as * = p ≤ 0.05, ** = p ≤ 0.01, *** = p ≤ 0.001 and **** = p ≤ 0.0001.

## 3 Results

### 3.1 Electrophilic STAT3 inhibitors block TrxR1 activity

To assess the electrophilic reactivity of the 23 STAT3 inhibitors we first investigated their ability to directly react with GSH using a DTNB reporter assay. Of the 23 compounds, 22 were easily seen to react with GSH over the 24 h timecourse of the experiment, as judged by decreased absorbance of liberated TNB^−^ anions upon addition of DTNB, suggesting that they can directly derivatize GSH *in vitro*. Notably, Stattic, Galiellalactone, and Disulfiram reacted rapidly with GSH in a timeframe of minutes. LLL12, Cpd188, Cucurbitacin I reacted over the course of several hours. Degrasyn, WP1066, Tyrphostin B42 were more resistant to GSH and the remaining compounds showed intermediate reactivity (Fig. S1). Auranofin, which did not give reliable output in this assay, has earlier been shown using other methods to easily react with GSH [38], suggesting that all compounds studied here have electrophilic properties and thus reactivity with GSH.

**Fig. 2:**
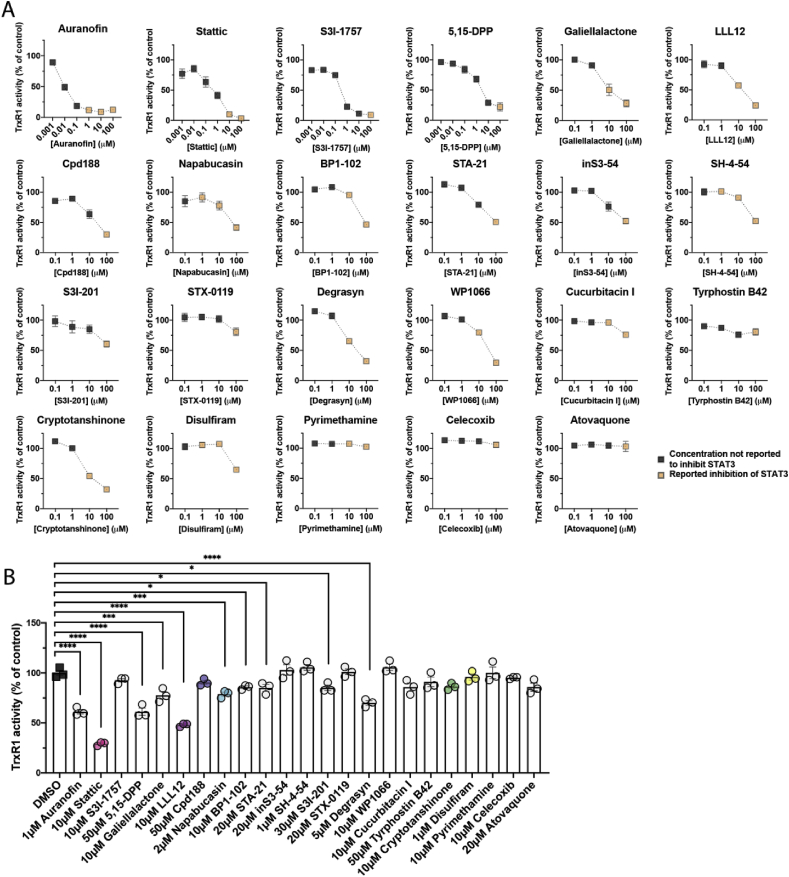
***In vitro* and cellular inhibition of TrxR1 activity by small molecule inhibitors of STAT3.** (A) TrxR1 (12.5 nM) activity was assessed *in vitro* after 40 min incubation with the compounds in presence of NADPH (250 μM), using an enzymatic DTNB reduction assay. (B) Selenium-supplemented A549 cells were incubated with compounds at the indicated concentration for 4 h before harvesting. Cellular TrxR1 activity was analyzed using the Trx1-linked insulin reduction endpoint assay. Color-coding was used to highlight compounds used in Fig. 3C–E. P-values are displayed as * = p ≤ 0.05, ** = p ≤ 0.01, *** = p ≤ 0.001 and **** = p ≤ 0.0001. (For interpretation of the references to colour in this figure legend, the reader is referred to the Web version of this article.)

We then specifically assessed the ability of the compounds to inhibit TrxR1 (Table 1 & Fig. 2A). Auranofin, Stattic, S3I-1757 demonstrated potent inhibition of TrxR1 activity, with full inhibition achieved at essentially stoichiometric concentrations. 5,15-DPP also demonstrated strong TrxR1 inhibitory activity with an IC_50_ value below 10 μM after 40 min incubation with 12.5 nM TrxR1. Moderate TrxR1 inhibitory activity was observed with Galiellalactone, LLL12, Cpd188, Napabucasin, BP1-102, STA-21, inS3-54, SH-4-54, Degrasyn, WP1066 and Cryptotanshinone, which in this assay all inhibited the enzyme by approximately 50% at concentrations between 10 and 100 μM.

Pyrimethamine, Celecoxib and Atovaquone did not affect TrxR1 reduction of DTNB at concentrations up to 100 μM, demonstrating lack of noticeable inhibition. Of the remaining compounds, S3I-201 and Disulfiram inhibited the enzyme less than 50% at 100 μM, while STX-0119, Cucurbitacin I, Tyrphostin B42 inhibited the enzyme less than 25%.

Next, we assessed cellular TrxR1 inhibition using human lung adenocarcinoma A549 cells, which are known to have high endogenous levels of TrxR1 [31]. In addition, the cells were supplemented with 100 nM sodium selenite, to ensure saturated levels of selenoprotein expression [45]. For these experiments we reasoned that choosing a short incubation time should allow for assessment of direct targeting of TrxR1 without confounding results due to differences in downstream effects, such as cytotoxicity or transcriptional responses, between the different compounds. Using the concentrations reported to inhibit STAT3 activity in cells (Table 1), Auranofin, Stattic, 5,15-DPP, Galiellalactone, LLL12, Napabucasin, BP1-102, STA-21, S3I-201 and Degrasyn all significantly lowered the TrxR1 activity in A549 cells after 4 h of treatment (Fig. 2B). While statistically significant for all these treatments, only the treatment with Stattic (10 μM) and LLL12 (10 μM) reduced the TrxR1 activity to less than 50% of DMSO treated controls under these conditions. It should be noted that TrxR1 activity was here assayed after a short time (4 h) of incubation and analyzing extracted cellular protein, suggesting that any detected inhibition of activity should be due to direct and irreversible inhibition of TrxR1, and not due to other unspecific effects of cytotoxicity.

### 3.2 A subset of compounds affects the thermal stability of TrxR1

**Fig. 3:**
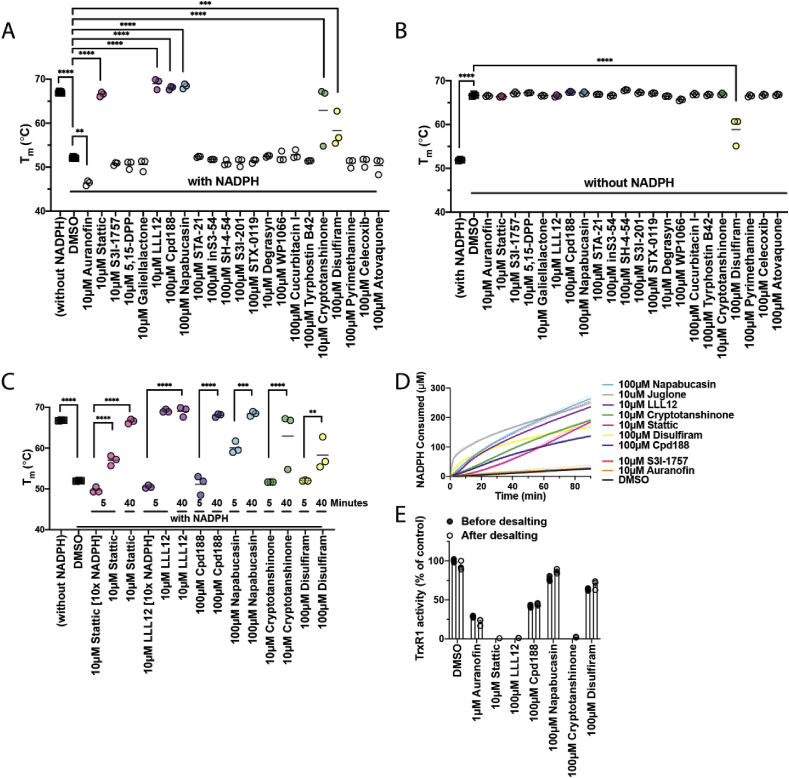
**Compounds that are both inhibitor and substrate of TrxR1 affect its thermal stability.** (A) TrxR1 (1 μM) was incubated for 40 min with compounds and NADPH (500 μM) before running DSF. Stattic, LLL12, Cpd188, Napabuscasin, Cryptotanshinone and Disulfiram thermally stabilize TrxR1. (B) TrxR1 (1 μM) was incubated for 40 min with only compounds in the absence of NADPH. Solely Disulfiram thermally destabilized TrxR1. (C) TrxR1 (1 μM) was incubated with compounds and NADPH [0.5 or 5 mM] for either 5 or 40 min. (D) NADPH consumption by TrxR1 (12.5 nM) during incubation of compounds tested in (C) over the course of 90 min. All compounds that thermally stabilize TrxR1 in DSF are also substrates of TrxR1. (E) TrxR1 (315 nM) was incubated *in vitro* for 40 min with compounds and NADPH (200 μM) before desalting. TrxR1 activity was measured with aliquots taken before and after desalting. All tested compounds that are substrates of TrxR1 also irreversibly inhibit Sec-dependent reduction of DTNB. T_m_ calculations of BP1-102 were excluded, because the background fluorescence from the compound interfered significantly with Sypro Orange™, which caused high fluorescent signal already at very low temperatures (Fig. S2). P-values are displayed as * = p ≤ 0.05, ** = p ≤ 0.01, *** = p ≤ 0.001 and **** = p ≤ 0.0001.

To investigate the interaction between the compounds and TrxR1 we investigated their abilities to alter the thermal stability of the enzyme using differential scanning fluorimetry (DSF). Oxidized TrxR1 was very stable with a T_m_ of 67 °C, while addition of NADPH to reduce the active sites of TrxR1 caused destabilization of the enzyme and a drop of T_m_ to 52.1 °C (Fig. 3A and B, Figs. S2–4). After 40 min of TrxR1 incubation with NADPH and compounds (10 or 100 μM, as indicated), Auranofin further destabilized reduced TrxR1 lowering its T_m_ to 46.4 °C. Stattic, LLL12, Cpd188 and Napabucasin instead stabilized reduced TrxR1 and increased its T_m_ to 66.5–69 °C, thus resembling the stability of oxidized TrxR1. Cryptotanshinone and Disulfiram stabilized reduced TrxR1 with its T_m_ increasing to 62.9 and 58.3 °C, respectively. Solely Disulfiram had an effect on the thermal stability of oxidized TrxR1 after 40 min of incubation, lowering its T_m_ to 58.8 °C, similar to the T_m_ of reduced TrxR1 treated with Disulfiram (Fig. 3B, Table 2, Fig. S2).

**Table 2:** Summarized effects on TrxR1 by explored STAT3 inhibitors.

Compound	Inhibition of purified TrxR1	Inhibition of cellular TrxR1	T_m_ with NADPH	T_m_ without NADPH	Substrate of TrxR1
Auranofin	++++	++	46.4 °C	66.6 °C	–
Stattic	+++	+++	66.6 °C	66.4 °C	+
S3I-1757	+++	–	50.8 °C	67.2 °C	–
5,15-DPP	++	++	50.6 °C	67.2 °C	–
Galiellalactone	++	+	50.6 °C	66.6 °C	–
LLL12	++	++	69.0 °C	66.5 °C	+
Cpd188	+	–	68.0 °C	67.4 °C	+
Napabucasin	+	+	68.4 °C	67.2 °C	+
BP1-102	+	+	–	–	–
STA-21	+	+	52.4 °C	66.9 °C	–
inS3-54	+	–	51.8 °C	66.6 °C	–
SH-4-54	+	–	51.0 °C	67.9 °C	–
S3I-201	+	+	51.2 °C	67.3 °C	–
STX-0119	(+)	–	51.4 °C	67.1 °C	–
Degrasyn (WP1130)	++	++	52.5 °C	66.5 °C	–
WP1066	+	–	52.4 °C	65.7 °C	–
Cucurbitacin I (JSI-124)	(+)	–	52.8 °C	66.9 °C	–
Tyrphostin B42 (AG490)	(+)	–	51.5 °C	66.8 °C	–
Cryptotanshinone	++	–	62.9 °C	66.9 °C	+
Disulfiram	+	–	58.3 °C	58.8 °C	+
Pyrimethamine	–	–	50.9 °C	66.5 °C	–
Celecoxib	–	–	51.2 °C	66.7 °C	–
Atovaquone	–	–	50.4 °C	66.8 °C	–

Underlining is indicative of a signficant change in T_m_ compared to DMSO-treated TrxR1.

### 3.3 Stabilizing compounds are also substrates of TrxR1

Since native oxidized TrxR1 is more stable than NADPH-reduced enzyme, and because several TrxR1 inhibitors can also be subversive substrates of the enzyme leading to NADPH consumption [[46], [47], [48], [49]], we next assessed whether the presence of NADPH or a shorter incubation time affected the impact on the TrxR1 thermostability by the stabilizing compounds. Indeed, additional experiments revealed that the T_m_ shift of reduced TrxR1 incubated with the stabilizing compounds, was modulated by shorter incubation time or addition of NADPH, which revealed a lower stability of the enzyme compatible with reduced (or inhibited) forms (Fig. 3C, Fig. S2). This result is compatible with the notion that Stattic, LLL12, Cpd188, Napabucasin, Cryptotanshinone and Disulfiram triggered a time-dependent consumption of NADPH leading to formation of oxidized forms of TrxR1 with higher T_m_, which was tested next.

The 6 compounds that stabilized reduced TrxR1 (Fig. 3A) in an NADPH- and time-dependent manner (Fig. 3C), were indeed found to act as substrates of the inhibited enzyme, i.e. inducing an increased consumption of NADPH (Fig. 3D). It should be noted that incubation of TrxR1 with these compounds also gave suprastoichiometric consumption of NADPH with regards to compound, with 10 μM of Stattic, LLL12 or Cryptotanshinone leading to consumption of more than 200 μM NADPH (Fig. 3D), thus showing redox cycling properties of the enzyme when inhibited by these compounds.

### 3.4 Some substrate compounds are also irreversible inhibitors of TrxR1

In order to analyze if the compounds found to be substrates of TrxR1 were also covalent inhibitors, we performed additional measurements of TrxR1 activity before and after desalting of the enzyme that had first been treated with NADPH and compounds for 40 min. All the compounds inhibited the Sec-dependent DTNB reduction by TrxR1 similarly before and after desalting, demonstrating that removal of unbound inhibitor from solution did not lead to a regain of TrxR1 activity. Napabucasin and Disulfiram had intermediate effects on TrxR1 under these experimental conditions, while the results with Stattic, LLL12, Cpd188 and Cryptotanshinone clearly suggested that these compounds are both covalent inhibitors of TrxR1 (Fig. 3E) and substrates of the otherwise inhibited forms of the enzyme (Fig. 3D).

## 4 Discussion

Due to the relative simplicity of assessing inhibition of STAT3 signaling in cells, many reports have identified compounds that block STAT3 phosphorylation and/or transcriptional activity. While many of such compounds are marketed in the literature as direct STAT3 binders, by utilizing molecular docking or biochemical methods, recent studies have shown that several of these inhibitors more likely act in a cellular context *via* indirect rather than direct mechanisms of STAT3 inhibition [8,15,16]. Therefore, the identification of direct cellular targets of small molecules that inhibit STAT3 functions in cells should be prioritized, if their mechanisms of action are to be fully understood. Furthermore, the distinct effects of STAT3 inhibition for any phenotypic experimental output could be distinguished more accurately, if additional (or genuine) cellular targets of STAT3 inhibitors are identified. In this respect, we recently identified a series of compounds that could bind STAT3 *in vitro*, as well as block cellular STAT3-dependent luciferase transcription. Using fluorescent probes, we determined that these compounds did not bind STAT3 in cells, but rather targeted TrxR1, which in-turn led to STAT3 oxidation and inactivation [15]. Here we therefore tested 23 previously reported small molecule inhibitors of STAT3 for any potential TrxR1 inhibitory activities. Three of these compounds potently inhibited TrxR1 *in vitro* at close to stoichiometric concentrations, and 10 compounds significantly inhibited TrxR1 activity in selenium-supplemented A549 cells after only 4 h of incubation. It is of course possible that additional compounds may inhibit TrxR1 activity in other cell exposure conditions, using different cell types or depending upon differences in cell uptake or metabolism, but we found it rather striking that as much as 10 out of 23 tested compounds designated as STAT3 inhibitors targeted TrxR1 in A549 cells within the short incubation time analyzed here.

We also identified that several of the compounds stabilized reduced TrxR1 *in vitro*, at least for Stattic, LLL12 and Cryptotanshinone, because they acted as redox cycling substrates with the otherwise covalently inhibited TrxR1. This type of effect with redox cycling of inhibited forms of TrxR1 was previously reported for several other compounds including juglone, dinitrohalobenzenes, curcumin, TRi-1, and indolin-2-one compounds [34,[49], [50], [51], [52]]. Forms of TrxR1 with a covalently targeted Sec-residue and inhibited normal TrxR1 activity, but with maintained capacity for redox cycling through an NADPH oxidase activity, were collectively coined SecTRAPs (Selenium compromised thioredoxin reductase-derived apoptotic proteins) and formation of SecTRAPs may yield therapeutic effects either through cytotoxicity against cancer cells or by Nrf2 activation in normal cells [17,26,34]. Our present results show that 6 out of 23 STAT3 inhibitors could directly provoke redox cycling with the otherwise inhibited TrxR1. Also, since as much as 10 of 23 tested STAT3 inhibitors inhibited cellular TrxR1 activity at concentrations reported to block STAT3 activity, the possibility that some of these compounds exert their STAT3 inhibitory activity *via* TrxR1 targeting should be considered. The effects on TrxR1 of the different STAT3 inhibitors studied here are summarized in Table 2.

The compounds that are described in this paper are a diverse set of inhibitors of cellular STAT3 activity, many of which hence also show inhibitory activity towards TrxR1. Auranofin, Stattic, Galiellalactone, BP1-102, SH-4-54, S3I-201, Degrasyn, WP1066 and Tyrphostin B42 have electrophilic moieties and the addition of antioxidants or reducing agents was reported to negate their inhibition of STAT3, thus further highlighting the importance of redox related effects of these compounds to inhibit STAT3 function [21,22,39,40,53,54]. In agreement with those findings and our present as well as recently published study [15], we suggest that direct inhibition of TrxR1 is likely to contribute to the STAT3 inhibitory activities of these compounds.

The mechanisms of STAT3 inhibition by STX-0119, Cucurbitacin I, Tyrphostin B42, Pyrimethamine, Celecoxib and Atovaquone have to be further investigated. We were unable to find any potent effect of these compounds on TrxR1 activity. Atovaquone is a patented drug to treat different parasites, targeting the mitochondrial electron transport chain [55]. Mitochondrial function and STAT3 are intricately linked, and recently two novel STAT3 inhibitors, OPB-51602 and OPB-111077, were found to be inhibitors of mitochondrial oxidative phosphorylation and able to resensitize cancer cells to tyrosine kinase inhibitors [8,56]. It is clear that there should be alternative mechanisms that can lead to cellular STAT3 inhibition. However, based upon the results presented here we suggest that TrxR1 targeting by small molecules should be a major and common mechanism leading to impaired STAT3 signaling.

## Declaration of competing interest

BDGP is listed as an inventor on a patent describing the STAT3 inhibitors BP1-102 and SH-4-054 (WO2013177534) and ESJA has several patents on specific TrxR1 inhibitors. The authors declare no other competing interests with regard to the presented data.
