# Supplementary material for: To inhibit TrxR1 is to inactivate STAT3–Inhibition of TrxR1 enzymatic function by STAT3 small molecule inhibitors
Source: Redox Biol. 2020 Jul 17;36:101646. doi: 10.1016/j.redox.2020.101646 (PMC7378686; doi:10.1016/j.redox.2020.101646)
Supplement: Multimedia component 1 [file mmc1.docx]

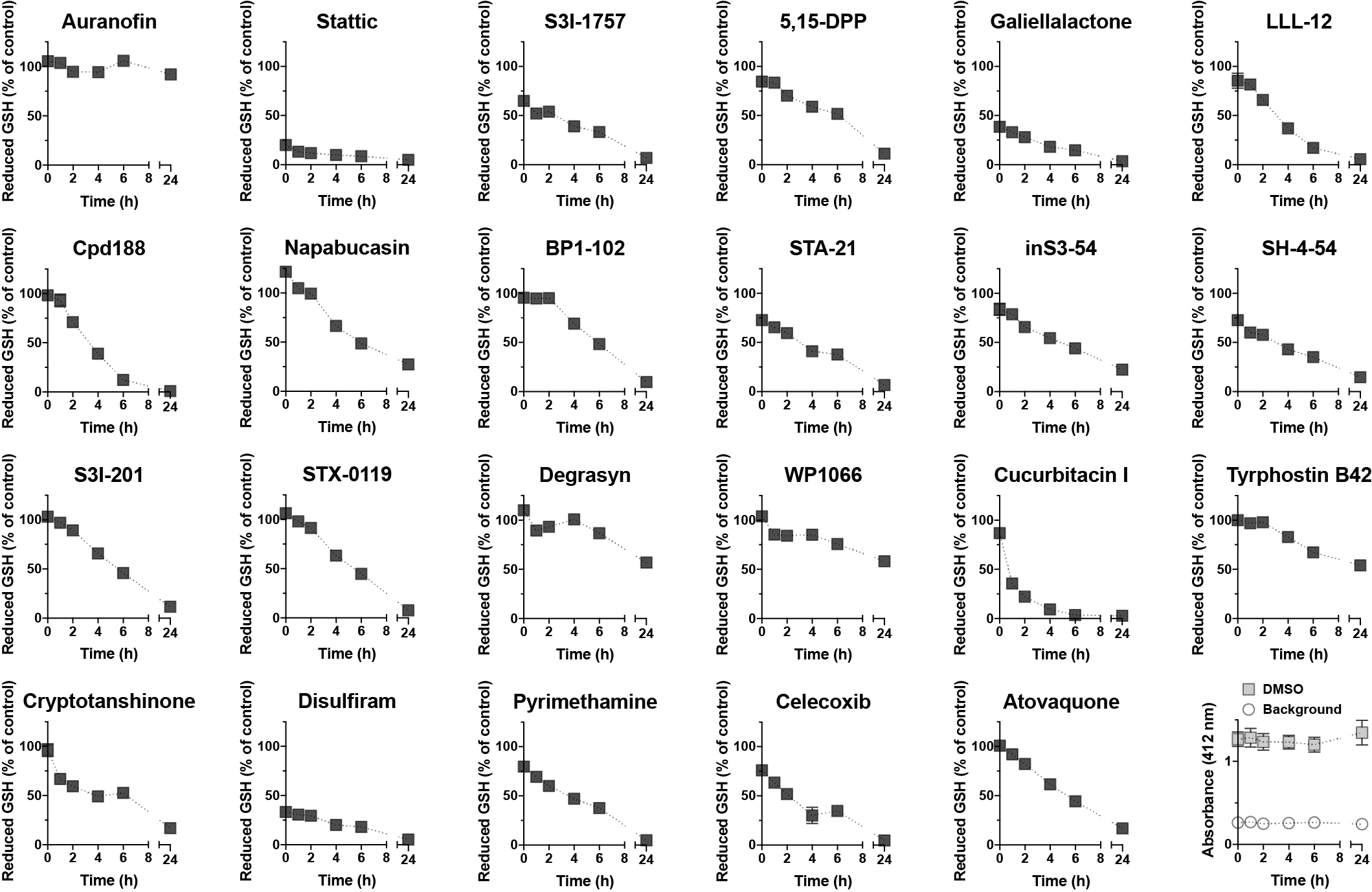


**Figure S1. Covalent reaction of compounds with GSH.** 175 μM compound was incubated with 175 μM GSH over a time course of 24 hours. 1 mM DTNB was added in order to assess the concentration of reduced GSH remaining in the reaction. Auranofin is known to react with GSH [38], we however did not detect any decrease in signal over time using our DTNB-based method with Auranofin, possibly due to reactivities of Auranofin-derived products with DTNB (scheme 1 of [38]).


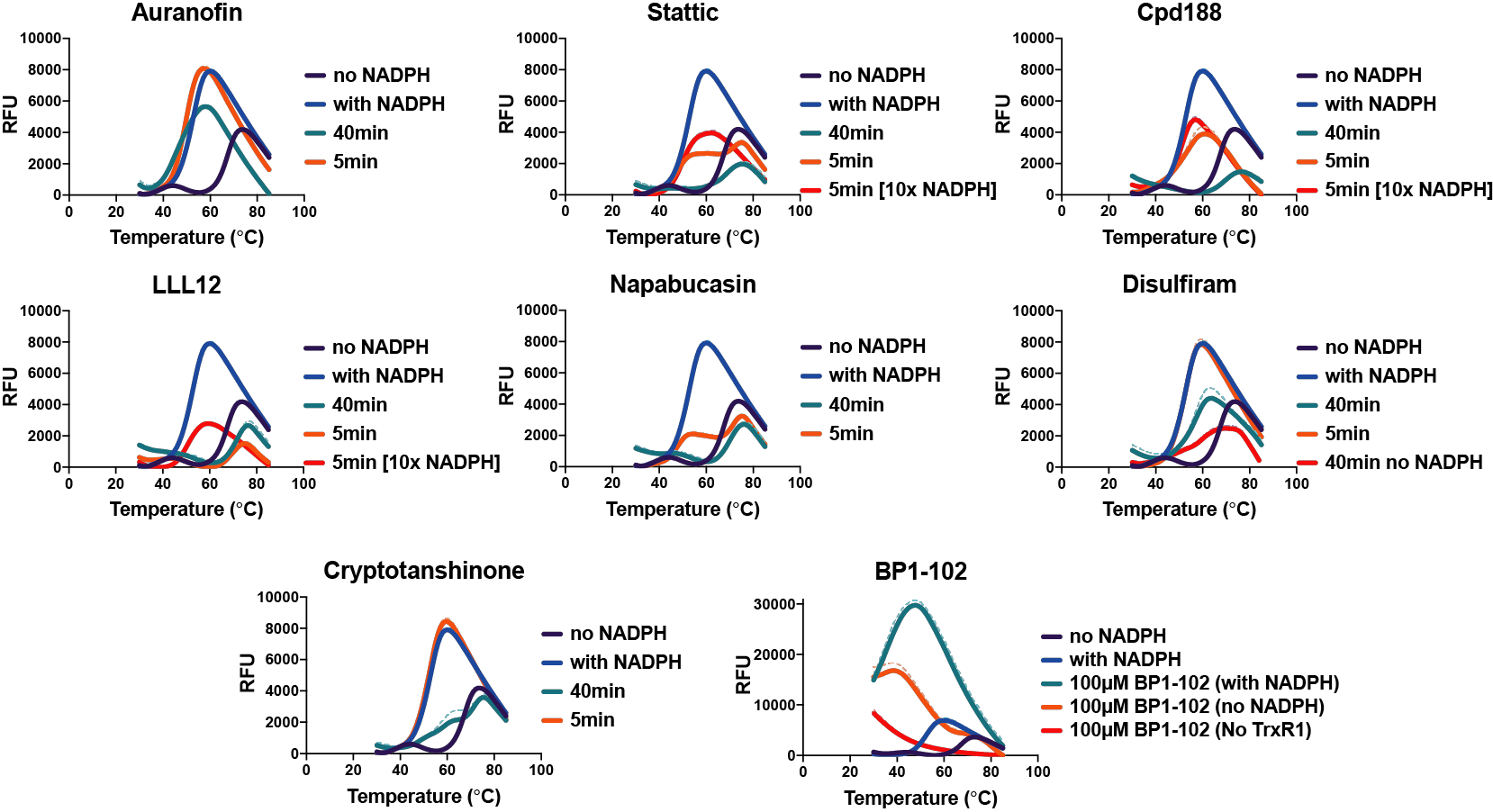


**Figure S2. Raw fluorescence curves of TrxR1 DSF with compounds that are both inhibitors and substrates of TrxR1.** Raw curves of Sypro Orange™ fluorescent signal measured from 30 to 85°C heating of TrxR1 with NADPH and either 5 or 40 minutes preincubation with compounds. BP1-102 was excluded from T_m_ interpretation because of high fluorescence interference of BP1-102 itself.

**
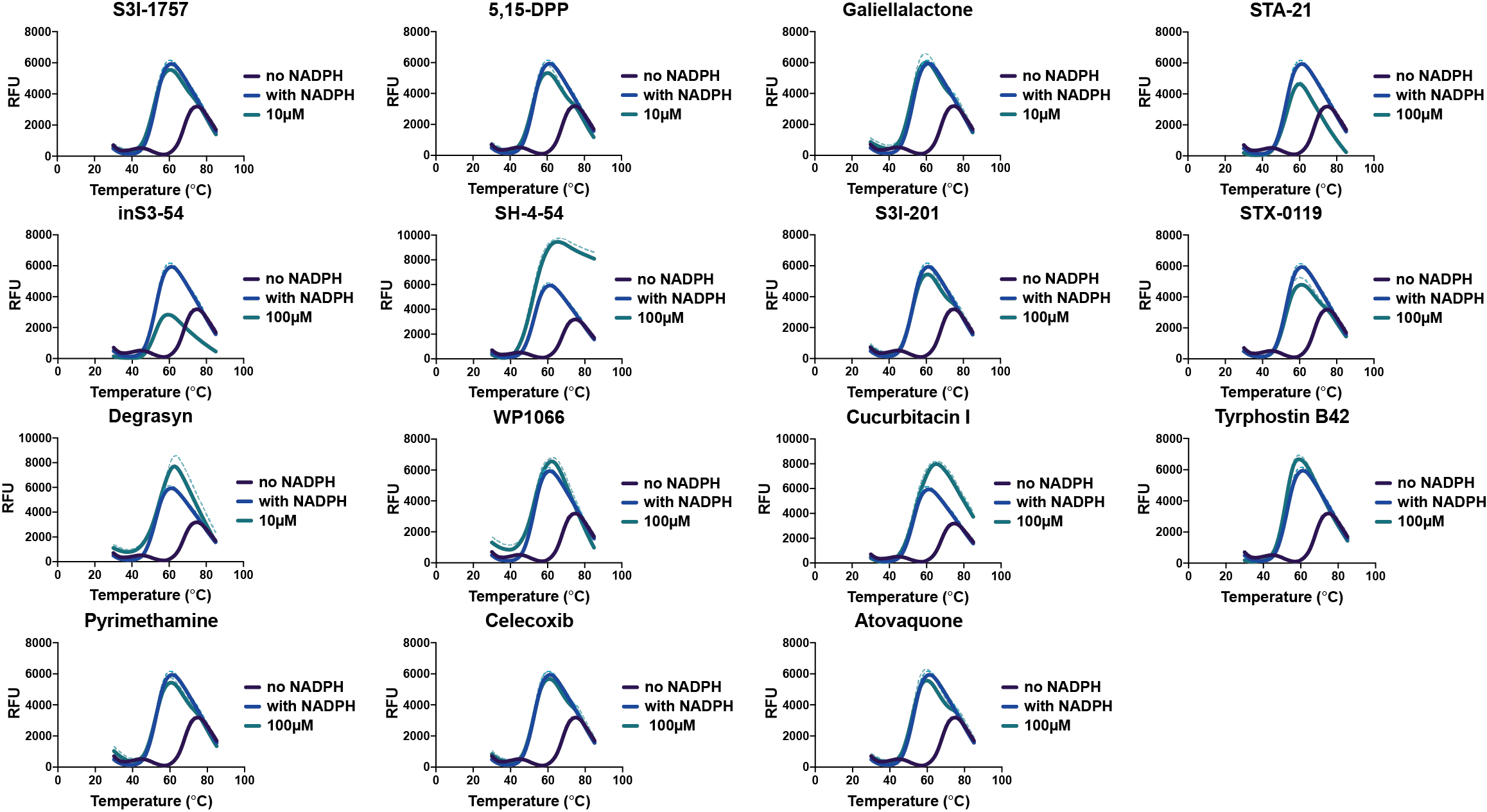
**

**Figure S3. DSF Raw fluorescence curves of TrxR1 with NADPH and compounds, that did not affect thermal stability.** Raw curves of Sypro Orange™ fluorescent signal measured from 30 to 85°C heating of TrxR1 with NADPH and compounds.


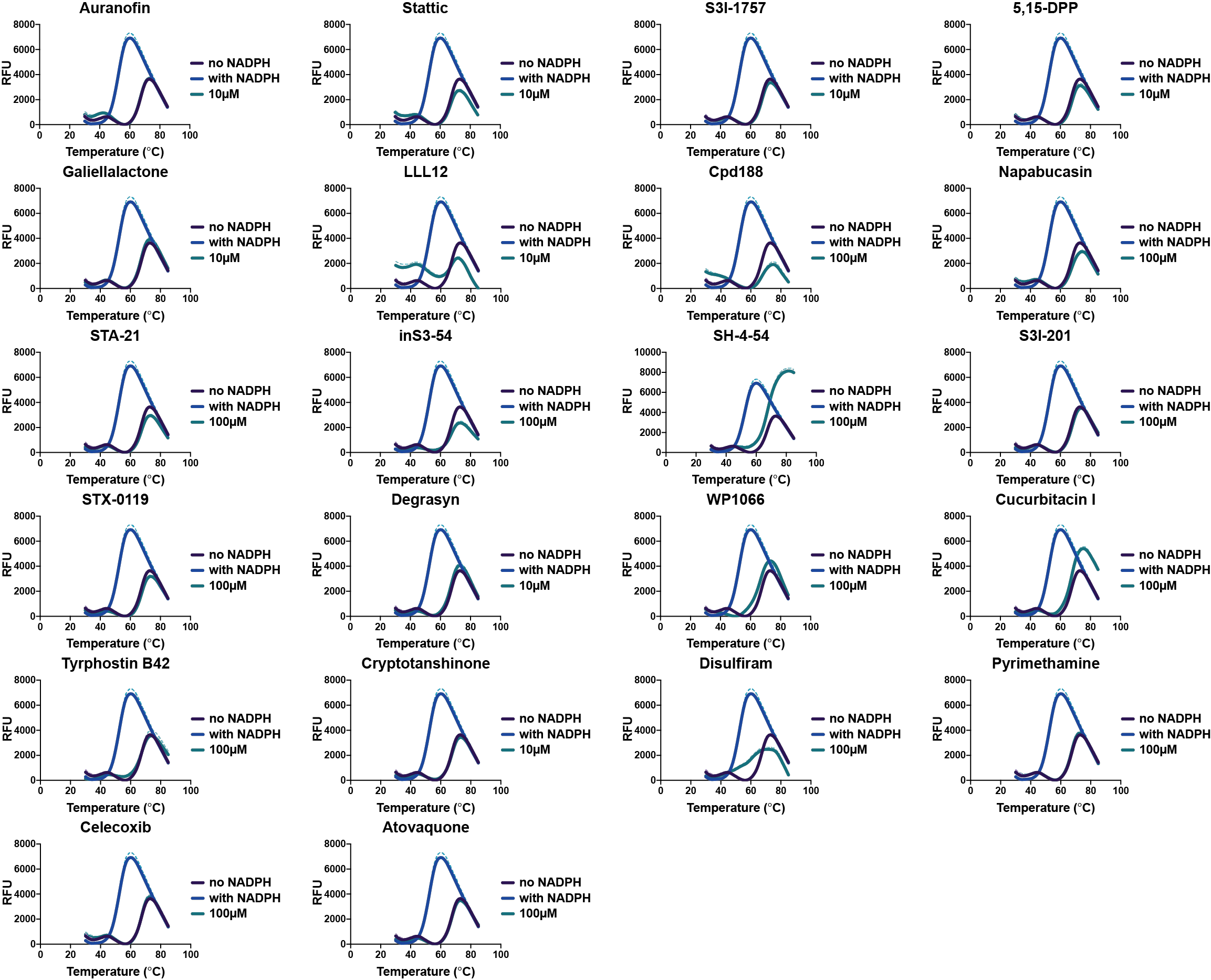


**Figure S4. DSF raw fluorescence curves of TrxR1 with only compounds.** Raw curves of Sypro Orange™ fluorescent signal measured from 30 to 85°C heating of TrxR1 with compounds. Only Disulfiram affected TrxR1 thermal stability, a clear alteration in curve shape and protein unfolding can be seen. LLL12 also appeared to affect the amount of fluorescence around 45-50°C, but this was not excluded from the T_m_ analysis.
